# Monitoring Voltage-Dependent Charge Displacement of *Shaker* B-IR K^+^ Ion Channels Using Radio Frequency Interrogation

**DOI:** 10.1371/journal.pone.0017363

**Published:** 2011-02-28

**Authors:** Sameera Dharia, Richard D. Rabbitt

**Affiliations:** Department of Bioengineering, University of Utah, Salt Lake City, Utah, United States of America; Instituto de Tecnologia Química e Biológica, Portugal

## Abstract

Here we introduce a new technique that probes voltage-dependent charge displacements of excitable membrane-bound proteins using extracellularly applied radio frequency (RF, 500 kHz) electric fields. *Xenopus* oocytes were used as a model cell for these experiments, and were injected with cRNA encoding *Shaker* B-IR (*Sh*B-IR) K^+^ ion channels to express large densities of this protein in the oocyte membranes. Two-electrode voltage clamp (TEVC) was applied to command whole-cell membrane potential and to measure channel-dependent membrane currents. Simultaneously, RF electric fields were applied to perturb the membrane potential about the TEVC level and to measure voltage-dependent RF displacement currents. *Sh*B-IR expressing oocytes showed significantly larger changes in RF displacement currents upon membrane depolarization than control oocytes. Voltage-dependent changes in RF displacement currents further increased in *Sh*B-IR expressing oocytes after ∼120 µM Cu^2+^ addition to the external bath. Cu^2+^ is known to bind to the *Sh*B-IR ion channel and inhibit *Shaker* K^+^ conductance, indicating that changes in the RF displacement current reported here were associated with RF vibration of the Cu^2+^-linked mobile domain of the *Sh*B-IR protein. Results demonstrate the use of extracellular RF electrodes to interrogate voltage-dependent movement of charged mobile protein domains — capabilities that might enable detection of small changes in charge distribution associated with integral membrane protein conformation and/or drug–protein interactions.

## Introduction

Techniques to monitor displacement currents in the protein-rich cell membrane have been used extensively in previous studies to examine ion-channel voltage-sensor movement [1], [2], [3], [4], [5], [6] and the piezoelectric-like behavior of transmembrane proteins [7], [8], [9]. These and similar studies record the magnitude and timing of charge displacement in the membrane to reveal the nature of voltage-dependent protein function and excitability in living cells. One technical challenge has been the electrical capacitance of the membrane lipid bilayer itself, which is in parallel with integral membrane proteins and obscures observations of protein-dependent electrical charge movements. This is compounded by the passive capacitance of the patch-clamp glass/quartz pipette that often limits the bandwidth of displacement current recording. In the present report, we introduce a new technique to monitor protein-dependent charge displacements by superimposing an extracellularly applied RF interrogation signal on top of a traditional voltage-clamp commanded membrane potential. This RF interrogation technique has its basis in electric impedance spectroscopy, which has been applied previously to probe membrane dielectric properties of isolated cells by measuring the electrical impedance between pairs or groups of extracellular electrodes [10], [11], [12], [13], [14], [15]. Here, we extend such RF dielectric measurements to study electrical charge displacement arising from electrically excited voltage-sensitive membrane-bound proteins.

**Figure 1:**
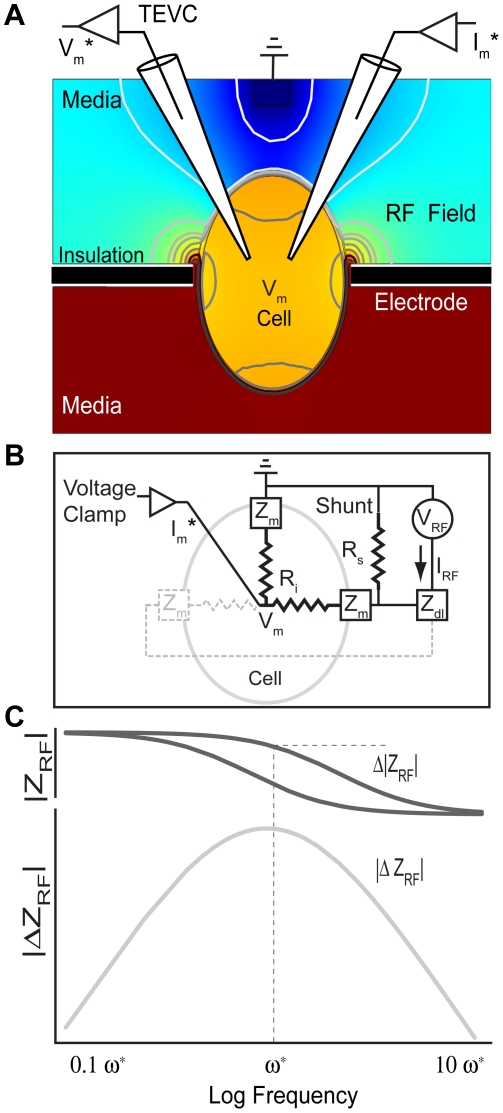
Set-up and circuit model. A) Changes in RF membrane impedance (|ΔZ_RF_|) during TEVC were measured by passing RF current from an electrode surrounding the meridian of the cell (black) to a ground electrode (media, above the cell). Contour lines and colors of the saggital cross-section of a cell in the recording chamber, shown here, illustrate the general spatial distribution of the RF electric potential expected based on the Maxwell equations for a passive cell under axisymmetric conditions (π/4 phase shown). B) A circuit model of the chamber including the shunt resistance (R_s_,), membrane impedance (Z_m_), intracellular resistance (R_i_), and electrode double layer (Z_dl_). C) Using the circuit model, the frequency-dependent RF impedance would change (Δ|Z_RF_|) with an increase or decrease in membrane capacitance — a change that would be most easily detectable at frequency ω* where the maxima of the |ΔZ_RF_| occurs. The present study reports changes in RF impedance |ΔZ_RF_| evoked by TEVC step changes in membrane potential.

*Xenopus* oocytes were used as a model cell for these experiments because they express a large amount of exogenous protein in their membrane; in these experiments, oocytes were transfected with the *Shaker* gene encoding *Sh*B-IR [15], [16], [17], [18], [19], [20], a well-characterized voltage-sensitive K^+^ ion channel with fast inactivation removed. A custom recording chamber was devised to allow for simultaneous two-electrode voltage clamp (TEVC) and RF impedance interrogation (Fig. 1A). Oocytes were placed individually into the center of an annular electrode that formed a tight conducting extracellular belt around the meridian of an oocyte (Fig. 1A). RF signals were passed from the annular belt (black electrode in Fig. 1A) to a single ground-wire electrode located above the oocyte. Quadrature lock-in amplification was used to measure the frequency-domain RF component of the voltage drop (*V_RF_*), current (*I_RF_*), and impedance (*Z_RF_ = V_RF_/I_RF_*) across the recording chamber. In this configuration, the RF component of the total current *I_RF_* was the sum of the conductive shunt current around the cell and the displacement current in the plasma membrane complex (Fig. 1B). Since the extracellular shunt conductance was much greater than the membrane conductance (Fig. 1B, *1* */R_s_* ≫*Real(1/Z_m_)*), RF impedance was not sensitive to voltage-dependent changes in membrane conductance and instead reflected voltage-dependent changes in membrane displacement currents [21]. Fig. 1C illustrates that a change in membrane displacement current would be detectable only over a limited frequency range due to the fact that shunt resistance (*R_s_*) would dominate at low frequencies and membrane capacitance would effectively short at high frequencies. In fact, previous work has shown extracellular shunt path estimation (frequencies <100 kHz) can be used to map cell shape in a recording chamber, and that frequencies above 3 MHz may be used to interrogate intracellular organelle distribution [21]. Examination of the impedance spectra for the specific system used in the present study revealed 500 kHz to be near the center of the sensitive range to maximize detection of membrane-related RF charge displacements [22].

Here we report changes in 500 kHz RF charge displacement evoked by step changes in membrane potential. TEVC was used to step the membrane potential (V_m_^*^) in the time domain, and to measure whole-cell currents (I_m_^*^, Fig. 1A). RF interrogating signals (*V_RF_*, *I_RF_*) were superimposed on top of the low-frequency TEVC commands. TEVC V_m_^*^ commands were low-pass filtered at 30 kHz so the TEVC signal contributed negligible power at the 500 kHz RF interrogation frequency. Similarly, TEVC I_m_^*^ signals were low-pass filtered to avoid the RF component. Lock-in amplification was used to extract electrical signals at the RF interrogation frequency. Data for *Sh*B-IR expressing oocytes were compared to control oocytes to examine the contributions of the expressed ion channel relative to endogenous proteins and lipids constituting the native oocyte membrane (control). In a subset of experiments, Cu^2+^ was applied to shift the voltage-dependence of *Sh*B-IR to more depolarized levels [23], [24] and to bind charge to the protein. Results show significant differences between voltage-dependent RF charge displacements in *Sh*B-IR expressing oocytes vs. controls. Differences are significant near the half-activation potential for the *Sh*B-IR channels, suggesting that RF data might correspond to ion-channel activity. Results further show that Cu^2+^ increased the magnitude of RF charge displacement while simultaneously shifting V_m_^*^ sensitivity to more depolarized levels on *Sh*B-IR expressing oocytes. This response was not as noticeable in control oocytes, indicating that the RF signal measured reflects RF vibration of the channel-protein–Cu^2+^ interaction. These results suggest that the RF-based technique introduced here could potentially supplement conventional membrane-biophysics studies, by monitoring RF mobility associated with membrane-bound charge distribution and charged molecule–protein (potentially drug–protein) interactions during voltage-clamp.

## Results

### Time-resolved changes in RF Impedance

**Figure 2:**
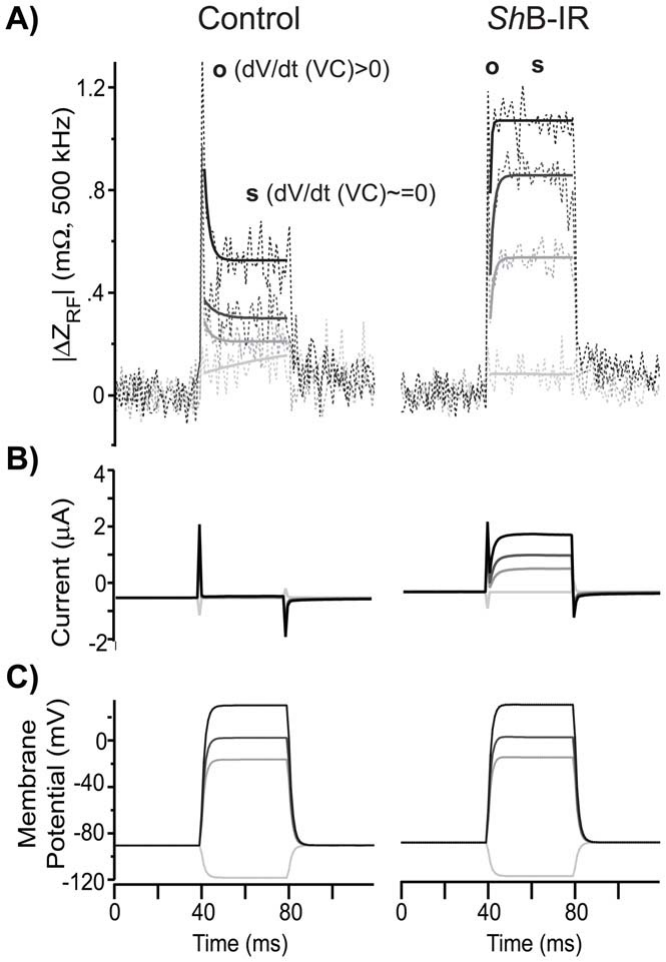
Temporally Resolved RF Measurements. A) RF impedance changes (|ΔZ_RF_|) measured during TEVC relative to the impedance at holding potential (−90 mV) in control oocytes, expressing endogenous proteins only (*n* = 10, left column), and *Sh*B-IR expressing oocytes (*n* = 9, right column). *Sh*B-IR expressing oocytes elicited a membrane-potential-dependent (V_m_^*^) RF response different than control oocytes. RF impedance changes were analyzed in two regions; the RF response during the onset of voltage-step (**o**, average |ΔZ_RF_|_o_ 0–1 ms after voltage step, dV_m_^*^/dt > 0) and the RF response after membrane potential achieved its command (steady-state) level (**s**, average |ΔZ_RF_|_s_ 5–35 ms after voltage step, dV_m_^*^/dt ≅ 0). B) TEVC current measurements were used to verify ion-channel expression and responses (leak current subtracted, capacitive transient unsubtracted) to C) whole-cell voltage-clamp.

Fig. 2A compares time-resolved changes in RF impedance |ΔZ_RF_| in control ooyctes expressing endogenous channels (avg. *n* = 10, left) to oocytes expressing *Sh*B-IR channels (avg. *n* = 9, right). Voltage-dependent RF charge displacement occurred in both cell expression systems, but differed in magnitude and temporal waveform between the control and *Sh*B-IR expressing oocytes. The left axis reports the magnitude of the change in impedance, |ΔZ_RF_| =  |Z_RF_- Z_0_|, where the reference, Z_0_, for each record was the average RF impedance 5–40 ms prior to the voltage step. Although signals were noisy in our apparatus, milliohm changes in |ΔZ_RF_| during depolarization were readily discernable from noise. In all cases, |ΔZ_RF_| consisted of an initial onset response when the rate of change of TEVC membrane potential was large (**o,** |ΔZ_RF_|_o_, dV_m_^*^/dt>0) and a steady-state response when TEVC membrane potential was approximately constant (**s**, |ΔZ_RF_|_s_, dV_m_^*^/dt ≅ 0). The RF onset response (**o**) occurred in the first millisecond after a voltage step was applied to the cell, and was similar in both the control and *Sh*B-IR expressing oocytes. Interestingly, the RF onset response was rectified and did not occur at the end of the voltage command step. Hence, the fast RF onset response was not causally related to the standard TEVC capacitive transient (current spikes in Fig. 2B). Steady state RF changes (**s**) 5–35 ms after the voltage step were also observed in both control and *Sh*B-IR oocytes, but were significantly larger in the *Sh*B-IR expressing oocytes at membrane potentials above −20 mV. This indicated a difference in RF response attributable to *Shaker* activation. Simultaneous TEVC recordings shown in the lower panels (Fig. 2B, current; Fig. 2C, voltage) were used to confirm expression and test whole-cell currents. Darker lines indicate increased levels of depolarization during the voltage step, commanded from a holding potential of −90 mV (Fig. 2C). TEVC whole-cell currents in the control condition (Fig. 2B, left) were small relative to the large currents in *Sh*B-IR expressing cells (Fig. 2B, right). Voltage sensitivity and sustained ionic currents reported here are typical for *Sh*B-IR cells with fast inactivation removed, and indicate successful protein expression and activity.

### Steady-state RF response for ShB-IR and Control Cells

**Figure 3:**
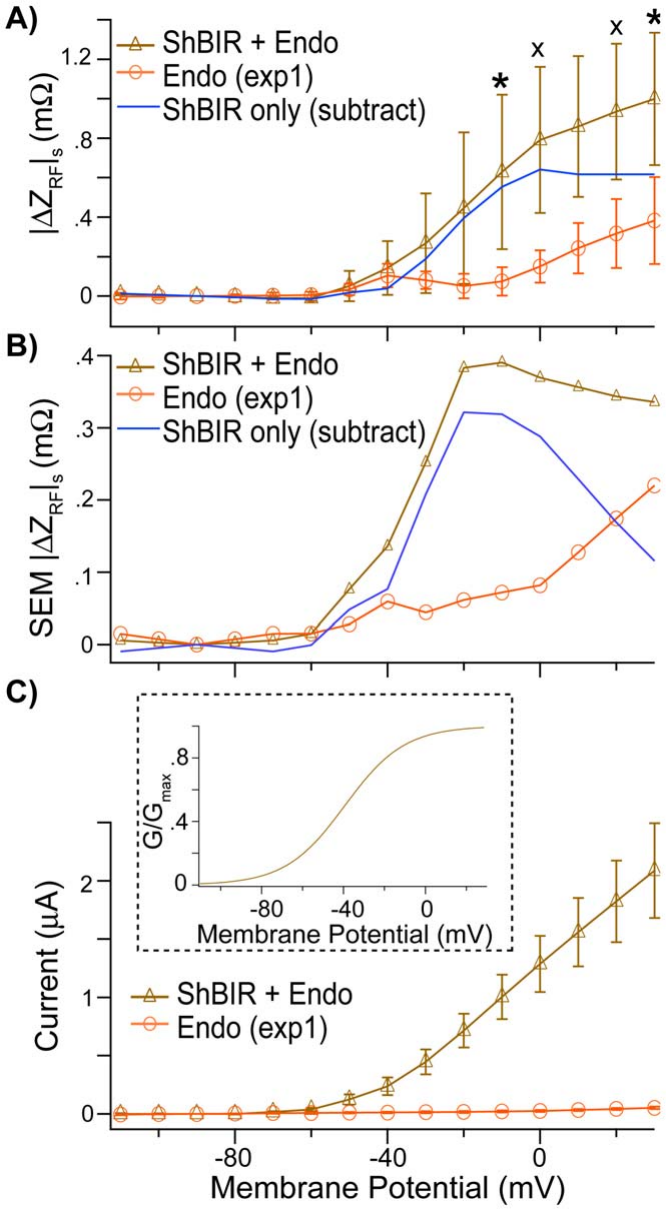
Steady-state RF response for *Sh*B-IR and Control Cells. A) Significant (^x^ *p* = .1, *p = .05) voltage-dependent differences in |ΔZ_RF_|_ s_ were observed between control oocytes, expressing endogenous proteins only (“Endo”, orange), and *Sh*B-IR expressing oocytes (express both endogenous and *Sh*B-IR proteins, “*Sh*B-IR + Endo”, brown). The “Endo” response was subtracted from the “*Sh*B-IR + Endo” response to estimate the isolated RF response from the *Sh*B-IR channels only (“*Sh*B-IR only”, blue). Error bars denote +/− standard errors of the mean (SEM). B) The SEM for the isolated *Sh*B-IR proteins (“*Sh*B-IR only”, blue) was also estimated by subtracting the SEM from the control oocytes (“Endo”, orange) from the SEM associated with the *Sh*B-IR expressing oocytes (“*Sh*B-IR + Endo”, brown). The SEM for isolated *Sh*B-IR expressing oocytes was largest near the half-activation potential for these ion channels. C) *Sh*B-IR channel expression and voltage-dependent whole-cell current was verified using TEVC, and this data was used to estimate *Sh*B-IR conductance (G/G_max_, inset).

To further examine differences between steady-state RF impedance in control (*n* = 10, “Endo”, orange) vs. *Sh*B-IR expressing oocytes (*n* = 9, “*Sh*B-IR + Endo”, brown), we averaged |ΔZ_RF_|_s_ 5–35 ms (see Methods) after the onset of voltage step (Methods), and plotted the result against the average membrane potential V_m_^*^ (Fig. 3A). *Sh*B-IR expressing oocytes include the exogenously expressed *Sh*B-IR proteins and endogenous membrane proteins and both contribute to the RF data. Averaged control data were subtracted from the *Sh*B-IR expressing oocyte data, to estimate the RF response associated with the *Sh*B-IR protein only (Fig. 3A, “*Sh*B-IR only”, blue line). Error bars denote standard errors of the mean (+/−, SEM), and are shown as a function of V_m_^*^ for *Sh*B-IR expressing oocytes and control oocytes in Fig. 3B. To demonstrate successful transfection, steady-state ionic currents are shown in Fig. 3C, and *Sh*B-IR channel conductance is shown in the inset. Like the averaged RF data, the SEM associated with the *Sh*B-IR protein only (Fig. 3B, “*Sh*B-IR only”, blue line) was estimated by subtracting the SEM associated with the *Sh*B-IR expressing oocytes at each membrane potential from the control oocytes SEM(s) (see Methods). Significant differences in |ΔZ_RF_|_s_ were found between *Shaker*-expressing oocytes and control oocytes at and above −10 mV (Fig. 3A), and differences are easily observable above the half-activation voltage for *Sh*B-IR channels (^x^ *p*<0.10, *p<0.05, Fig. 3A). Interestingly, the RF *Sh*B-IR-only data (Fig. 3A, blue line) and the corresponding *Sh*B-IR conductance data (shown in Fig. 3C) have a linear correlation coefficient of .94 (and a correlation coefficient of .99 in the voltage range of −45 mV to 2 mV, when the *Sh*B-IR data increase from 5–95% of their final value), indicating a positive association between channel activity and the measured RF response. Furthermore, unlike the control oocytes that showed increased SEM for large V_m_^*^, the *Sh*B-IR-only SEM (Fig. 3B, blue line) showed the largest value near the half-activation voltage, and standard errors for the *Sh*B-IR expressing oocytes were comparable to baseline values when the ensemble of channels in the membrane were predominantly closed or open. These results are consistent with voltage-dependent SEM arising from probabilistic conformational state of the *Shaker* ion-channels expressed in the membrane.

### Copper treatment and steady-state ShB-IR RF response

**Figure 4:**
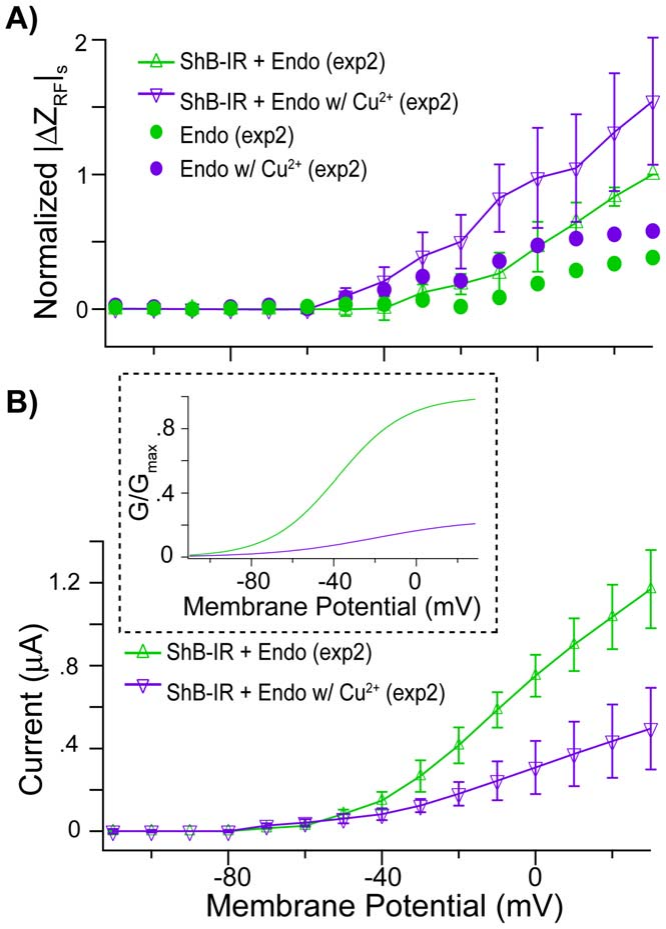
Copper treatment and steady-state *Sh*B-IR RF response. A) Voltage-dependent differences in |ΔZ_RF_|_s_ were observed between *Shaker* expressing oocytes (“*Sh*B-IR + Endo”, green line) and the same cells exposed to ∼120 µM Cu^2+^ (purple line). A similar effect was apparent, albeit to a lesser extent, for control cells before (“Endo”, green markers)/after Cu^2+^ treatment (purple markers). Error bars denote +/− standard errors of the mean (SEM). B) Even though RF charge displacements increased in Cu^2+^-exposed *Sh*B-IR expressing oocytes, TEVC whole-cell current decreased showing that Cu^2+^ successfully blocked the channels (channel conductance shown as inset).

A subset of *Sh*B-IR expressing oocytes (*n* = 4) were treated with Cu^2+^, a K^+^ ion-channel modulator, and normalized |ΔZ_RF_|_s_ were determined for these oocytes before and after Cu^2+^ treatment (exp2). Results are shown in Fig. 4A as a function of steady-state membrane potential (V_m_^*^). Data from each cell were normalized to its RF impedance at +30 mV for the nontreated *Sh*B-IR expressing oocytes, to permit comparisons between cells before/after copper treatment (see Methods). As expected, Cu^2+^ application greatly reduced the *Sh*B-IR current (I_m_^*^) in the voltage-range where *Sh*B-IR channels activate (Fig. 4B). *Sh*B-IR channel conductance before and after copper addition are shown in the inset (Fig. 4B). The change in RF impedance after copper treatment was larger than the untreated condition (Fig. 4A). To examine statistical significance for this relatively small population, data above −60 mV (see Methods) were pooled together. Pooled data showed a statistically significant difference between nontreated vs. Cu^2+^-treated *Sh*B-IR expressing oocytes (*p* = .04, U = 1007, Total Points = 80, normalized median-values of the nontreated/treated *Sh*B-IR expressing oocytes are .36 and .90, respectively; see Methods). Results from control cells (*n* = 2, Fig. 4A) are scaled by the ratio of the control cell to *Sh*B-IR cell data at +30 mV (shown in Fig. 3) to enable inter-cellular comparison, after normalizing the data from each control cell to its nontreated |ΔZ_RF_|_s_ measured at +30 mV (see Methods). A change in the normalized |ΔZ_RF_|_s_ is noticeable in the control cells after Cu^2+^ application (above −60 mV), indicating that Cu^2+^ might non-specifically interact with the endogeneous oocyte membrane in addition to the known effect of binding the *Sh*B-IR channels. While non-specific Cu^2+^ likely contributed to the change in RF response before/after Cu^2+^ addition, the effect in *Sh*B-IR expressing oocytes was much larger, suggesting that the RF also detected Cu^2+^ interaction specifically with the *Shaker* channels. As depolarization level increased, the effect of the bound Cu^2+^ increased. At 30 mV, the Cu^2+^-treated *Sh*B-IR expressing oocytes exhibited a response approximately 1.5 times that of the non-treated *Sh*B-IR expressing oocytes, even though the ionic current (TEVC) was approximately one fifth of the non-treated cells.

### RF Onset Response in ShB-IR Expressing Oocytes

**Figure 5:**
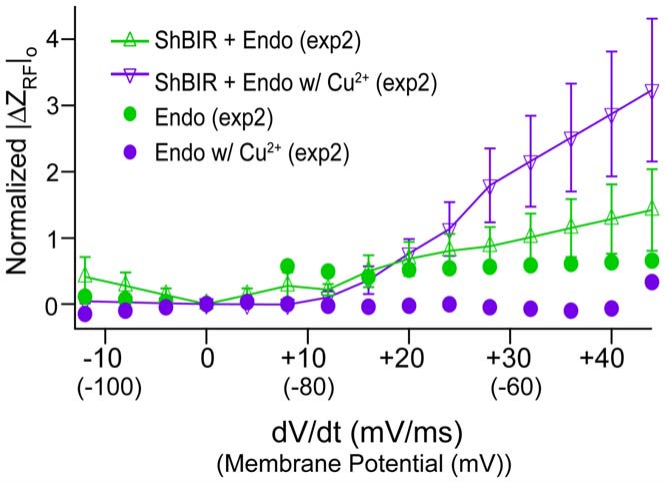
Onset RF Response in *Sh*B-IR Expressing Oocytes. Changes in RF impedance during the onset of voltage-clamp (|ΔZ_RF_|_o,_ 0–1 ms after whole-cell depolarization) were slightly depressed in control oocytes with the addition of Cu^2+^ (Cu^2+^-free - green markers, Cu^2+^ addition - purple markers), but were significantly greater in *Sh*B-IR expressing oocytes (Cu^2+^-free - green line, Cu^2+^ addition - purple line).

RF onset responses, occurring during the rise of V_m_^*^ (when dV_m_^*^/dt was maximum), exhibited trends different than those seen in steady state. Fig. 5 plots the onset response |ΔZ_RF_|_o_, averaged 0–1 ms after voltage command was applied (see Methods) vs. the rate of change of membrane potential (dV_m_^*^/dt) and membrane potential (parenthetically noted below the values of dV_m_^*^/dt). Cell data have been normalized using the same values in Fig. 4A, to allow for comparisons between transient and steady-state RF results (see Methods). The fast onset recorded in *Sh*B-IR expressing oocytes and controls both increased with the magnitude of the membrane potential change, but there were no statistically significant differences due to expression of K^+^ channels (*p*>.1, data not shown). Onset RF charge displacements in control oocytes treated with Cu^2+^ slightly decreased as voltage-step increased (Fig. 5, filled circles), and in contrast to *Sh*B-IR expressing oocytes, the average onset response of controls treated with Cu^2+^ was smaller than the non-treated cells. Cu^2+^-treated *Sh*B-IR expressing oocytes pooled for all voltage commands > −60 mV exhibited significantly larger onset responses than non-treated *Sh*B-IR expressing oocytes (*p* = .005, U = 1087, Total Points  =  80, median values untreated/treated *Sh*B-IR expressing oocytes of 1.0/1.4, respectively). Interestingly, increases in the average onset |ΔZ_RF_|_o_ for Cu^2+^ treated *Sh*B-IR expressing oocytes were about 1.5 times greater than those for Cu^2+^ treated *Sh*B-IR oocytes in the steady state (|ΔZ_RF_|_s_) for membrane potentials >−60 mV. One hypothesis that might explain the Cu^2+^-dependent onset response is that Cu^2+^ might have stabilized a transitional state in the ion channel during rapid membrane depolarization that momentarily affected the charge distribution on the protein, thereby enhancing the momentary RF charge displacement. The onset response was not detectable for -dV_m_^*^/dt at the end of the command pulse (see Fig. 2A), showing a rectification in |ΔZ_RF_| quite distinct from dV_m_^*^/dt driven linear capacitive transients (present in Fig. 2B).

## Discussion

Here we introduce a new technique to monitor voltage-dependent charge displacement associated with *Sh*B-IR K^+^ ion channels using RF electric fields during TEVC. The RF electric field provided a vibrational force that resulted in measurable RF charge displacement on a cell membrane complex caused by vibrating membrane-bound charges. The 500 kHz RF was superimposed on top of low-frequency TEVC (<30 kHz) allowing the RF to continuously interrogate changes in charge displacement mobility during whole-cell depolarization. Changes in RF impedance, |ΔZ_RF_|, were recorded every 20 µs, and provided a nearly instantaneous view of the effective dielectric behavior of the protein–membrane–ion complex. Voltage-dependent changes in |ΔZ_RF_| were averaged into millisecond bins (Fig. 2) and showed time-resolved differences for *Sh*B-IR expressing oocytes relative to controls, demonstrating the sensitivity of this approach to detect RF vibrational motion associated with ion-channel-specific charge displacements.

Analysis of RF charge displacements revealed significant differences in |ΔZ_RF_|_s_ between *Sh*B-IR expressing oocytes and controls at depolarized membrane potentials (Fig. 3A). Differences were largest above the half-activation potential for the *Shaker* channels used here. The *Sh*B-IR channels have fast inactivation removed, and as such, significant changes in steady-state RF vibration demonstrate that the open conformation of *Sh*B-IR channels have measurably different effective dielectric behavior than the closed conformation. It is important to note that this difference was present at steady-state and did not reflect the classical gating-charge movement that typically occurs for these channels ∼0–2 ms after voltage command [4], [25]. Instead, voltage-dependent changes in RF charge displacement |ΔZ_RF_|_s_ appear to reflect changes in mobile charge displacement magnitude within the transmembrane RF electric field and/or stiffness associated with the open vs. closed state of the *Shaker* protein (measured during steady-state). Consistent with this, the steady-state RF impedance associated with the *Sh*B-IR proteins had a positive correlation with channel conductance (cf. blue curve in Fig. 3A and 3C inset). This hypothesis is further supported by the fact that the SEM for the *Sh*B-IR proteins (*Sh*B-IR expressing oocytes – control oocytes) was greatest at the channel's half-activation potential, and smaller when the protein was completely hyperpolarized (all channels closed) or depolarized (all channels open). This same trend in SEM was not observed in control oocytes, and can only be explained if the observed changes in effective dielectric were due to charge vibrations specific to the *Shaker* protein.

Cu^2+^ addition to the bath significantly changed steady-state RF impedance |ΔZ_RF_|_s_ in *Sh*B-IR expressing oocytes relative to controls. In particular, data indicated that while a portion of the steady-state RF response may be caused by non-specific binding of Cu^2+^ to the cell membrane, Cu^2+^ also binds specifically to the *Sh*B-IR protein and increases RF –evoked charge displacement during membrane depolarization. Analysis of the *Sh*B-IR expressing oocyte data showed that differences between Cu^2+^-treated and non-treated cells were present at membrane potentials as low as −50 mV and increased with TEVC depolarization (Fig. 4A). The increase in RF response caused by Cu^2+^–*Shaker* interaction occured even as ion-channel conduction current decreased (Fig. 4B). One possibility is that Cu^2+^ binding to the ion channel enhanced |ΔZ_RF_|_s_ by increasing the effective charge moving within the transmembrane electric field and this thereby enhanced the signal arising from the RF-evoked protein vibration.

In addition to steady-state changes in RF charge displacement, rapid onset responses |ΔZ_RF_|_o_ were detected during positive depolarizing steps (*dV_m_/dt>0*) in both *Sh*B-IR expressing and control oocytes (see Fig. 2). Interestingly, the onset response was large and easily observable in both cell expression systems (control and *Sh*B-IR) only for positive *dV_m_/dt*. Hence, the onset RF data are not directly related to the traditional capacitive transient current evoked during voltage clamp. The present data were not adequate to analyze the decay time constant of the RF onset response in the *Sh*B-IR expressing cells due to the superimposition of the large RF response associated with the *Sh*B-IR channel activation. The magnitude of the transient response in both control and *Sh*B-IR expressing oocytes was comparable, indicating the importance of endogenous mechanisms contributing to the RF onset response (data not shown here). This shows that the RF onset displacement currents measured here are not caused by the gating charge movement of *Shaker* channels. This was not surprising, due to the slow voltage clamp rise time in the present experiments. A faster voltage-clamp and RF recording system might allow future investigation of voltage-sensor displacement via RF interrogation. Even with the slow voltage-clamp used here, the onset response |ΔZ_RF_|_o_ increased significantly in *Sh*B-IR expressing oocytes treated with Cu^2+^ (Fig. 5). Data for *Shaker*-expressing cells in Fig. 5 were normalized to the same values as the steady-state figure, and show that |ΔZ_RF_|_o_ in the Cu^2+^-treated cells was 1.5 times as high as the values shown for the same copper-treated cells in the steady-state. One possible explanation for the large value of |ΔZ_RF_|_o_ could be that the membrane-ion complex undergoes a non-equilibrium polarization when *dV_m_/dt* is positive, compelling a brief period of enhanced dipole mobility. If true, this effect may be amplified or augmented by Cu^2+^–membrane–*Sh*B-IR interactions.

Although interpretation of the RF results can be challenging on the surface, this type of data cannot be obtained by traditional electrophysiological techniques and may prove useful in understanding membrane-bound protein dynamics. Present results show that the method can be used to track time- and voltage-dependent changes in RF charge displacement associated with membrane-bound ion-channel state/conformation and electrostatic binding of charged compounds. As such, the technique supplements conventional electrophysiological techniques and is suitable to examine voltage-dependent membrane-bound protein conformations and potentially also pharmacological interactions.

## Methods

### Oocyte Injection

*Xenopus* oocytes were isolated at the University of Utah's Cardiovascular Research and Training Institute. cRNA encoding *Sh*B-IR was injected into oocytes 1–2 days before experimentation, to allow ample time for channel expression. Oocytes were stored in Superbarths oocyte media (88 mM NaCl, 1 mM KCl, 0.41 mM CaCl_2_, 0.33 mM Ca(NO_3_)_2_, 1 mM MgSO_4_, 2.4 mM NaHCO_3_, 10 mM HEPES, 1 mM pyruvate, and 50 µg/ml gentamicin, titrated using NaOH to a pH of 7.4) at 17° Celsius and ionic currents measured using two-electrode voltage clamp (TEVC) were used to verify channel expression.

### Experimental Set-Up

An oocyte (∼1.2 mm diameter) was positioned in a 1.2 mm recording chamber filled with Super Barth's Oocyte media surrounded by a 18 µm thick, gold-plated copper, effectively annular electrode (ORFLO, Woodinville, WA). Oocyte positioning and electrode stabilization were facilitated by a polycarbonate interface; the electrode was stabilized by two separate plates of the polycarbonate interface, and an oocyte was loaded into the recording chamber through a bowl-shaped well [22]. Two glass micropipettes, used for two-electrode voltage clamp and filled with 3M KCl, were carefully placed in the oocyte using a 2X magnification lens on an upright microscope (AxioTech, Zeiss, Thornwood, NY). Each micropipette had a 0.5–2 MΩ access resistance, and was driven by a two-electrode amplifier (AxoClamp2B, Molecular Devices, Sunnyvale, CA) that controlled voltage-measuring (HS-2Ax1LU, Molecular Devices, Sunnyvale, CA) and current-injecting headstages (HS-2Ax10MGU, Molecular Devices, Sunnyvale, CA). An Ag/AgCl ground electrode was placed approximately 5 mm above the oocyte and immersed in media near the top of the recording chamber. This ground was common for both the RF recordings and TEVC. Photographs taken of the oocyte before and after experimentation were used to confirm minimal oocyte movement during recording (Q-Color3, Olympus, Center Valley, PA).

A∼120 µM Cu^2+^ solution (Copper ICP/DCP standard solution 10,000 µg/mL Cu^2+^in 2% HNO_3_, Sigma Aldrich, St. Louis, MO) was applied as an ion channel modulator to a small subset of the oocytes involved in experimentation. The Cu^2+^ solution was manually pipetted directly above the cell. The concentration of Cu^2+^ added was approximate, as it depended on the volume of Superbarths oocyte media in the recording chamber (∼1.25 mL).

### Recording

A 500 kHz radio frequency was passed from the gold-plated annular electrode to the ground electrode [22]. Simultaneously, TEVC was used to control membrane potential (V_m_^*^, single-pole low pass filter, 30 kHz). Oocytes were held at −90 mV (holding potential) for 360 ms, and a voltage step was then applied for 40 ms. Nine different voltage steps were applied to the oocyte (−120 mV, −60 mV, −40 mV, −30 mV, −20 mV, −10 mV, 0 mV, 10 mV, 40 mV), and are collectively referred to as a voltage train. Voltage trains were applied to each cell 50 times. Voltage-clamp was automated using a software package designed for this purpose (Patchmaster, HEKA Inst., Bellmore, NY; Igor Pro, Wave Metrics, OR).

RF voltage drop across the recording chamber (*V_RF_*) and RF current (*I_RF_*) were monitored using an onboard reference impedance to track excitable membrane impedance changes (*Z_RF_ = V_RF_/I_RF_*). Head-stage amplification (OPA356, Texas Instruments, Dallas, TX), a 100 kHz high pass filter (48 dB/Oct Bessel HPF; SIM965, Stanford Research Systems, Sunnyvale, CA) and lock-in amplification (SR844, Stanford Research Systems, Sunnyvale, CA) were used to extract the RF component applied during TEVC. Voltage-clamp current and commands were low-pass filtered at <30 kHz to further ensure minimal cross-talk between the TEVC and RF recording systems. The quadrature lock-in signals, as well as TEVC ionic current, membrane potential and applied command potential were recorded through a 16-bit A/D converter (ITC-1600, HEKA Inst. Bellmore, NY). Patch-clamp software (Patchmaster, HEKA Inst., Bellmore, NY) sampled and saved data from each A/D channel at a rate of 20 µs. This voltage-clamp software in conjunction with the signal averaging time on the lock-in were the rate limiting factors for temporal resolution during RF interrogation. The technique, however, given the Nyquist frequency of the RF, has the potential to resolve events down to 4 µs.

### Data Analysis

Data from individual oocytes were averaged over 50 command presentations (each consisting of 9 voltage levels, as described above). TEVC currents were leak compensated for each cell by assuming that currents measured during hyperpolarization were predominately caused by leak, and that these currents changed linearly with voltage. Leak-subtracted TEVC data for each oocyte were binned in 1 ms intervals in the time domain, and used to estimate *Sh*B-IR channel conductances (Ohm's law: I_TEVC_^*^  =  G*_SH_*_B-IR_ (V_m_^*^-E_rev_)). Capacitive transients were not subtracted. TEVC currents were plotted as a function of membrane potential, and current data (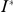) collected at V_m_^*^ > −10 mV were linearly interpolated to 0 to estimate E_rev_. Error between I and (V- E_rev_) was then minimized by providing the best fits to a sigmoidal conductance (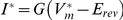, where 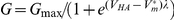; 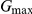
is the maximum whole-cell conductance of *Sh*B-IR expressing oocytes; V_HA_ is the half activation potential; λ is the rate of conductance rise). Channel conductance values were normalized to the maximum conductance value (G/G_max_), and are shown as insets in Fig. 3 and 4.

Magnitude change in RF impedance (|ΔZ_RF_|, 500 kHz interrogation frequency) for each cell was calculated relative to the average RF impedance 5–35 ms before voltage command (holding potential ∼ = −90 mV). This was done in one millisecond bins. Temporally based comparisons of control *vs. Sh*B-IR cells and Cu^2+^-treated *vs.* non-treated oocytes (like in Fig. 2) were made by averaging data (TEVC current, TEVC voltage, |ΔZ_RF_|) across control oocytes (*n* = 10), *Sh*B-IR expressing oocytes (consist of both endogenous and *Sh*B-IR protein, *n* = 9), *Sh*B-IR expressing oocytes before/after Cu^2+^ treatment (*n* = 4) and control oocytes before/after Cu^2+^ treatment (*n* = 2). Voltage data were used to align voltage steps from individual cells in time to minimize jitter during averaging.

To facilitate comparisons across cells, the average value of |ΔZ_RF_| was calculated for each cell at every voltage-step 1) 5–35 ms after the voltage step had been applied (|ΔZ_RF_|_s_, dV_m_^*^/dt (voltage clamp) ≅ 0, “steady state response”) and 2) 0–1 ms after the voltage step had been applied (|ΔZ_RF_|_o_, dV_m_^*^/dt (voltage clamp) ≠ 0, “onset response”). To compensate for slight differences in TEVC membrane potential between individual cells, normalized RF data were linearly interpolated between voltages for each cell before averaging. Steady-state data were interpolated at the following membrane potential levels {−110 mV, −100 mV, −90 mV, −80 mV, −70 mV, −60 mV, −50 mV, −40 mV, −30 mV, −20 mV, −10 mV, 0 mV, 10 mV, 20 mV, 30 mV}. Onset response was plotted as a function of dV_m_^*^/dt, and results were interpolated to the following values {−12 mV/ms, −8 mV/ms, −4 mV/ms, 0 mV/ms, 4 mV/ms, 8 mV/ms, 12 mV/ms, 16 mV/ms, 20 mV/ms, 24 mV/ms, 28 mV/ms, 32 mV/ms, 36 mV/ms, 40 mV/ms, 44 mV/ms}.

The sample sizes used in the *Sh*B-IR cell (*n* = 9) vs. control cell (*n* = 10) experiment (exp1) allowed for direct (non-normalized) comparison between both data sets, as it was assumed that the approximate shunt path associated with the *Sh*B-IR expressing oocytes and control oocytes was approximately equal across the populations (Fig. 3). Standard errors of the mean were calculated for both control and *Sh*B-IR expressing oocytes at specified membrane potentials (−110 mV, −100 mV, −90 mV, −80 mV, −70 mV, −60 mV, −50 mV, −40 mV, −30 mV, −20 mV, −10 mV, 0 mV, 10 mV, 20 mV, 30 mV). The *Sh*B-IR expressing oocytes consisted of RF responses from both endogenous proteins on the oocyte membrane and the exogenously expressed *Sh*B-IR protein (referred to as “*Sh*B-IR + Endo” in Fig. 3). The *Sh*B-IR and endogenous protein responses were assumed to be approximately independent, and the averaged control data (endogenous protein only, “Endo”) was subtracted from the averaged *Sh*B-IR expressing oocyte data (“*Sh*B-IR + Endo”, Fig. 3) to estimate the RF response associated with the *Sh*B-IR-protein only (“*Sh*B-IR only”, blue line, Fig. 3). Similarly, the standard error of the mean associated with the *Sh*B-IR protein-only was calculated by subtracting the SEM associated with control oocytes from the SEM associated with the *Sh*B-IR expressing oocytes (blue line, Fig. 3b).

The effects of Cu^2+^ application on both steady-state and transient RF impedance were compared for *Sh*B-IR expressing oocytes (*n* = 4) and control oocytes (*n* = 2, exp2). The small sample size prevented voltage-dependent population statistics for these controls, but we were able to make comparisons within cells. Data for each cell before/after copper treatment were normalized to the steady-state RF impedance value of that specific cell before Cu^2+^ treatment at +30 mV. After normalization, data for each group (Control-untreated/treated, *Sh*B-IR-untreated/treated) were averaged together, and the effects of Cu^2+^ before/after treatment were compared within each cell expression system. Control data (both before/after copper treatment) were scaled by the ratio between the control cells and *Sh*B-IR cells at +30 mV from the previous experiment (larger sample sizes, Fig. 3). This scaling enabled comparisons between the control cell data and the *Sh*B-IR cell data before/after copper treatment. Standard errors of the mean were calculated for *Sh*B-IR expressing oocytes at specified membrane potentials (−110 mV, −100 mV, −90 mV, −80 mV, −70 mV, −60 mV, −50 mV, −40 mV, −30 mV, −20 mV, −10 mV, 0 mV, 10 mV, 20 mV, 30 mV). SEM was not calculated for control cells, due to the small (<3) sample size.

In exp1, data at each voltage for both the control and *Sh*B-IR expressing cells were not normally distributed, due to differences in shunt path between cells and differences in exogenous protein expression level on the *Sh*B-IR expressing oocytes. As such, a Mann-Whitney nonparametric statistical test was used to compare control vs. *Sh*B-IR expressing oocyte data (exp1) at each of the sampled voltage levels (the median of the sample values were slight lower than the averages shown in Fig. 3A, although the trends were similar). The null hypothesis (H_0_) for this comparison was that the RF data for the *Sh*B-IR cells before are the same as the RF data for control oocytes at a specified voltage (V_m_^*^, membrane potential) level. The linear correlation coefficient between *Sh*B-IR conductance and the subtracted *Sh*B-IR-only RF response was calculated both between -110 mV and 30 mV and between −45 and 2 mV (the regime where the RF *Sh*B-IR-only response increased from 5–95% of its final value).

In exp2, the sample size of the *Sh*B-IR expressing oocytes (*n* = 4) before/after Cu^2+^ treatment was too small to apply the Mann-Whitney test at every voltage level (requires a total sample size of 10). Instead, all the normalized data sampled from cells above −60 mV (where the channel begins to activate) were pooled, and these pooled samples were compared before/after copper treatment using the Mann-Whitney test (the pooled voltage levels for each group of cells was the same). This allowed for a significance test between the two treatment groups of cells, with the null hypothesis (H_0_) that the RF data for the *Sh*B-IR cells before copper treatment are the same as the RF data for the *Sh*B-IR expressing oocytes after copper treatment.
